# When bushfires and the climate crisis become the new normal

**DOI:** 10.1016/j.lanwpc.2025.101503

**Published:** 2025-02-28

**Authors:** The Lancet Regional Health – Western Pacific

The unprecedented scale of the recent wildfires in California in January have brought the climate crisis into focus again. In many ways, the disaster feels strikingly similar to the Black Summer of 2019—one of the most catastrophic fire seasons in Australian history. Bushfires are neither new nor rare in Australia. As of 5th February, residents in Victoria are similarly being urged to abandon their homes and evacuate from another out-of-control blaze as the world’s attention remains fixed on the USA. The rest of the world might assume that Australia has recovered from the Black Summer, but the reality is that bushfire cycles still repeat yearly and become a part of the seasonal rhythm. Scientists have even forecast up to a 70% increase in the number of days with high-to-extreme fire risk by 2050, implying that climate consequences will only get more regular and imminent, and set the standard for what is now considered the new normal.

The historically hot and dry climate has made bushfires an intrinsic component of the Australian ecosystem. While climate change was not the direct cause for past fires, it has exacerbated the frequency, intensity, and duration of fires. Rising temperatures and altered rainfalls have led to faster growth of flammable vegetation. The evaporated moisture in the air also boosts the likelihood of lightning strikes to ignite dry wood. Between 1967 and 2013, major Australian bushfires caused at least 8000 injuries and 433 deaths, equivalent to AUD$4·7 billion worth of direct economic loss, yet the adverse effects on health were barely calculated. With bushfire smoke spreading thousands of kilometres, the levels of PM_2·5_ and PM_10_, the components of which are often more toxic than other sources of pollution, could reach four times higher than those recommended by WHO, resulting in respiratory and cardiovascular distress, eye irritation, and adverse perinatal outcomes. The Black Summer of 2019 accounted for over 6000 emergency department visits particularly in regions with lower socioeconomic status. Studies also revealed that repeated exposure to climate disasters worsened mental health outcomes, and the symptoms related to depression, anxiety, and post-traumatic stress disorder persisted 3 years after fires. The long-lasting physical and psychological repercussions are often overlooked, but evident.

In the face of recurring threats, one might question how Australia adapted from its devastating experience to advise the world. Since the deadly Canberra fires in 2003, efforts were seen at the state level, including the set-up of asset protection zones between properties and vegetation, new building standards to withstand heat, pre-fire season controlled burns and targeted grazing to reduce fuel, and spatial forecasting models. At the national level, the federal government released the first National Climate Resilience and Adaptation Strategy in 2015 to prioritise actions to mitigate the climate crisis and founded the National Emergency Management Agency in 2022 to coordinate bushfire management. Despite encouraging initiatives, they have received criticism that health was not always centred in the proposed strategies. Plans for smoke exposure management seemed less developed than the efforts on risk reduction and warning systems, yet the health burden from smoke often outweigh those directly from fires. It was not until 2023 that the National Climate and Health Strategy was announced to emphasise health consequences. To date, however, there remains a lack of risk-based health measures targeted to subgroups at risk, and a lack of studies on the effectiveness and cost-effectiveness of the policy actions on health outcomes.

These initiatives taken are some positive steps despite the shortcomings. However, the speed of gathering efforts into adaptation, rescue, and recovery has been increasingly outpaced by the consequences of the climate crisis itself in recent years. Under the new normal, for example, the fire seasons in Australia have evolved to be longer. This leaves a shorter window for controlled fuel reduction burn, which can only take place in certain weather conditions. Lengthier fire seasons have also coupled with the increased vulnerability to large-scale wildfires in other places, such as what we saw in California, to cause overlapping fire seasons between the southern and northern hemispheres and hinder multinational shared rescue assistance. Although another megafire could strike again at anytime, the community need for victims from the Black Summer is far from over—Neighbourhood Houses in Victoria are at risk of closure owing to debt and unsustainable recovery funds in the post-pandemic era, and are not in a position to boost community readiness ahead of the next disaster. Moreover, living under the constant threat of losing lives and properties, residents in fire-prone regions could face anticipatory anxiety, but it is unclear if such a pre-disaster psychological toll will be addressed in the next national plans timely.

In contrast to passive adaptation to escalating climate impacts, it can be more efficient to revisit the root cause of the climate crisis and tackle it at its source. As with many countries in the Western Pacific, Australia is highly susceptible to climate extremes, but this country continues to be one of the biggest coal producers in the world and lagged behind most Organization for Economic Cooperation and Development countries in incentivising the popularisation of electric vehicles. In the USA, the withdrawal from the Paris Climate Agreement immediately after President Trump took office on 20th January, and his political shift towards domestic fossil fuel production, also underscore how national self-interests can derail global progress towards fighting the climate crisis. It is crucial to have every country come together again for a new dialogue that prioritises the urgency of the climate crisis over individual agendas.

Without collective commitment, climate consequences—either manifested as bushfires or not—will only accelerate and offset the adaptation efforts made so far. To reverse the new normal, future global actions must keep up with the pace of the evolving challenges, and more importantly, put health centre stage.

